# The Low-Wage Essential Worker: Occupational Concerns and Needs in the COVID-19 Pandemic—A Round Table

**DOI:** 10.1177/2165079920988682

**Published:** 2021-03-31

**Authors:** Susan Gallagher, Asha Roy, Sandra J. Domeracki, Todd Mohrmann, Vicki Missar, Janet Jule, Shreela Sharma, Ryan DeWitt

**Affiliations:** 1 Celebration Institute, Inc; 2 Northwell Health; 3 San Francisco Veterans Affairs Medical Center; 4 Dynamic Training; 5 Aon Risk Services, Inc., of New York; 6 Kaiser Permanente; 7 The University of Texas; 8 Blue Sky Farms

**Keywords:** underserved, low-wage worker, disease transmission, COVID-19, occupational health and safety, essential worker, social justice

## Abstract

**Background:**

Planning occupational health and wellness services and support directed toward low-wage, essential workers in the COVID-19 pandemic has posed a number of challenges across work settings. This article explores the concerns and needs of low-wage essential workers as understood by experts in the field.

**Methods:**

Leading experts in the areas of occupational health and safety, risk management, insurance, and professional education/training were identified and invited to participate in a Round Table discussion. Questions posed to experts were based on literature that addressed COVID-19, essential workers, low-wage workers, infection transmission, education/training, and social justice.

**Findings:**

Experts agreed that special considerations must be in place to address the concerns and needs of the low-wage essential worker. These special considerations should address not only the worker’s occupational experience but their family and home environment, fears and anxieties, and the economic impact of the COVID-19 restrictions and requirements.

**Conclusion/Application to practice:**

The occupational health professional is a key resource to employers charged with addressing the concerns and needs of low-wage, essential workers during the COVID-19 pandemic.

## Background

Although the term *essential worker* varies slightly from state to state, the United States Department of Homeland Security describes essential workers as those individuals who conduct a range of operations and services that are typically essential to the operations of critical infrastructure (National Conference of State Legislatures, 2020). Critical infrastructure is then described as a large, umbrella term encompassing sectors from energy to defense to agriculture (Cybersecurity and Infrastructure Security Agency, 2020). Examples of an essential worker include teachers, farmworkers, healthcare workers, food processing and distribution workers, police officers, and communication workers. Not all categories of essential workers are low-wage; however, many are. For instance, healthcare aids, restaurant workers, housekeeping services, and farm and factory workers are just a few examples of workers who are needed and considered essential, and also earn a low wage (Fusaro & Shaefer, 2016).

In February 2020, before the COVID-19 pandemic, many low-wage workers were already living in an uncertain financial situation. In the United States, 22% of the workforce, approximately 31 million people, earned a median wage below US$11.19 per hour (Garfield et al., 2020). Over a quarter of low-wage workers lived in a household without a full-time worker in the family, and more than half were in a family with the total family income below 200% of poverty, which is US$26,200 for a family of four in 2020. Low-wage workers reportedly expressed insecurities associated with day-to-day financial concerns even before COVID-19. One third indicated that they were very or moderately worried about making their monthly financial commitments. Low-wage workers also were likely to experience food insecurity, with 15% meeting federal definitions of low (9%) or very low (6%) food security. Furthermore, many lower income households reported US$492 or less in liquid assets (Garfield et al., 2020).

In a study conducted by the Rapid Assessment of Pandemic Impact - Early Childhood (RAPID-EC) team at the University of Oregon, workers who earned the lowest wages were the least likely to have paid sick leave, health insurance, life insurance, or other aspects of an economic safety net (RAPID-EC Project Team—University of Oregon, 2020). For this category of worker, missing work to recover from an illness or injury may pose an economic hardship. Healthcare costs can be economically overwhelming. In the event of death, few economic resources are available to family members for burial and other costs. For these millions of Americans, even a few missed shifts can leave families struggling to pay for the basics, such as rent or groceries. There is a lack of options available to low-wage, essential workers to cope with the pandemic, as many lack access to unemployment benefits, emergency paid sick or family leave or health insurance. (Flores & Padilla, 2020).

Low-wage essential workers have shown to be at great risk for COVID-19 infection. From an infection transmission perspective, in a recent study, Flores and Padilla (2020) report California’s 58 counties and the U.S. Census Bureau found a strong relationship between low-wage work and COVID-19 positive test rates. Conditions in the agricultural work setting place workers at high risk for COVID-19 transmission. For instance, Flores and Padilla (2020) reported that recent outbreaks among pistachio plant workers in Wasco, meatpacking plant workers in Hanford, or field-workers in Oxnard and Imperial County reveal the rapid spread of COVID-19 in rural communities with high proportions of low-wage workers. Other industries have also suffered from high rates of transmission, particularly accommodations (hospitality) and food services.

This Round Table, which includes input from content experts, explores the concerns and needs of the low-wage worker who is categorically defined as essential. Literature that was either referred to or inferred is summarized in a review of resources and references for the occupational health professional (see Table 1).

**Table 1.** A Review of Resources and References for the Occupational Health Professional About the Impact of COVID-19 on Low-Wage Essential Workers

Berude and Bateman (2020): Thousands of workers are feeling the economic impact of the COVID-19 recession. This paper, sponsored by the Brookings Institute, outlines the many job categories affected directly or indirectly by the pandemic. “Everyday Words for Public Health Communication” (2016): This resource offers specific strategies for better written and spoken communication by using straightforward language that resonates with the general public. Garfield et al. (2020): As the title “Double Jeopardy: Low Wage Workers at Risk for Health and Financial Implications of COVID-19” suggests, this paper, sponsored by the Kaiser Family Foundation, offers a comprehensive description of the health and financial concerns of the person who is a low-wage worker in the face of the COVID-19 pandemic. A must read for researchers. “Health Literacy Universal Precautions Toolkit” (2020): The Agency for Healthcare Research and Quality (AHRQ) describes the meaning of health literacy and outlines the steps to communication and readability. Specific downloadable tools and resources are available to readers. Kinder and Ross (2020): This paper, sponsored by the Brookings Institute, argues that policy makers need to take a closer look at inequities as they play out in the lives of low-wage essential workers in the COVID-19 pandemic.

## Methods

Experts selected to participate in this Round Table bring a wide variety of education, training, and experience. Risk manager/analysts, a professional health educator/trainer, senior leaders, and occupational health professionals were invited to participate. Representatives from the dairy, child care, food bank, and healthcare industries were included. Dairy professionals and representatives from the food bank understand challenges associated with the potential for limited physical distancing because of work performed in close quarters. Risk managers and insurance analysts are trained in identifying the data analytics and targeting risk mitigation from an organizational policy perspective. Professional educator/trainers understand the wide variety of background learners bring to the classroom and offer a unique perspective on training all learners, including the low-wage essential worker. Recognizing the impact this topic poses to the occupational health professional is outlined, and interprofessional ideas are presented and debated.

## Findings

***Most would agree that the COVID-19 pandemic, as it unfolded, came as a surprise to many Americans living in the US. This may be particularly true of low-wage workers, who are described as living on an hourly rate that does not meet a living wage, and essential workers, who categorically are described by the 2013 Essential Services Act as employees who perform work involving the safety of human life and the protection of property. States have the ability to refine this designation, but generally an essential worker, in this context, is a worker who is unable to perform their job remotely or in the safety of their own home. In your opinion, what are some of the real-life or unforeseeable challenges pertaining to injury/illness, and social or safety risks among essential low-wage workers?***

Asha Roy (AR): One of the most significant issues with this group is that, for many, English is not the first language. A good portion of the essential worker population consists of immigrant workers. Any education and training we provide going forward must account for this possible variance in comprehension. Furthermore, education must be customized to the level of understanding observed within this population. During the first wave, which happened so quickly here in New York, we did not have the opportunity to address this gap. Now we can take measures to account for this and customize education and training. This gap was one of the real-life, unforeseeable challenges that affected the learning needs of essential, low-wage workers.

Ryan DeWitt (RD): In the dairy industry, like many hourly jobs, workers often work numerous shifts at a variety of different plants/dairies. This practice increases occupational exposure. For example, if there is an outbreak in a nearby dairy where our employees occasionally work, then our dairy is at risk for a similar outbreak. We read about this in the meatpacking industry, restaurants, nursing homes, and elsewhere. This common practice poses a safety risk to our essential dairy workers. We understood that limiting this practice posed unforeseeable financial hardship to our workers. The local dairies collaborated for a solution that met the needs of both employers and employees. As a solution, they agreed that each dairy would offer employees as many shifts as possible as long as the employee agreed to work at a single dairy, rather than multiple dairies. We believe this will continue to prevent transmissions from one site to another.

Shreela Sharma (SS): We find that low-wage workers may be uninsured or underinsured, and hence, illness will not only affect their regular wages and employment but also additional medical costs that can be incurred. Furthermore, these financial hardships trickle into other social issues such as food insecurity or housing insecurity, which can further place the individual more vulnerable to physical disease and emotional stress. Both physical disease and emotional stress can lead to illness requiring time away from work. Time away from work creates a cyclical impact pushing the individual further into financial hardship. This is especially concerning when the individual lacks economic reserves.

Vicki Missar (VM): One issue with low-wage workers as it relates to workers’ compensation benefits is that many workers have multiple jobs so it is difficult to ascertain root cause of the injury or illness. This is the situation Mr. DeWitt described and it is not unusual. In addition, I agree with Dr. Sharma, time away from work is a financial burden on all workers who lose income, but especially low-wage workers who lack economic reserves. Employers often forget this as policies are written that preclude the employee from working without provision for income. This leads to employees attempting to work while infected.

Janet Jule (JJ): The COVID-19 pandemic has posed several challenges to low-wage workers in the healthcare environment. One unforeseeable situation was the level of anxiety among our support service employees in housekeeping or dietary. Sanitation was expected to be performed differently and more frequently. Housekeepers were expected to clean and sanitize high touch surfaces such as elevator buttons and doorknobs more frequently. They were also responsible for refilling all hand degermers in the work setting. There were training challenges as described by Ms. Roy earlier.

There are family stresses as well. For instance, essential workers do not have the option to work remotely. Those workers who had school-aged children found there were child care needs as well as challenges with the distance learning programs. Juggling care for children is often overlooked by employers. In addition, some workers experienced a high level of anxiety being in the hospital setting for fear of exposure to COVID-19 and then taking it home to their families.

***Dr. Jule mentioned the level of anxiety that is associated with the fear of exposure. Have mental health concerns become a factor? How is this manifesting in the work setting?***

JJ: Mental health concerns have become a factor in the low-wage workers’ overall situation. Increased anxiety due to uncertainty about the future and instability of employment are major contributors to this population’s stress. Those who serve as sole providers in their households must balance work hours with their responsibilities at home. This can lead to overwhelming fatigue and irritability. It is not unusual for increased stress to manifest itself in aggressiveness at the workplace. This can further lead to friction with colleagues and supervisors or even the threat of workplace violence.

Sandra Domeracki (SD): We found this as well. During support groups, we learned that aging workers in the housekeeping service had concerns when being assigned to COVID-19 units. Their stress levels increased significantly as they are more aware of their increased risk of illness severity when contracting COVID-19 due to their age and numerous comorbidities.

AR: Fear of exposure and fear of infecting others, especially their loved ones, is constant stress within this population. Going back to the example of this population being immigrant workers, some have joint families and have older parents living at home. An additional source of stress is not getting the time to grieve their coworker’s demise due to COVID-19. We have seen some deaths within this population but never truly explored resources provided for grieving. Imagine losing a close friend you have been working with for years and the next day having to show up to work and having to work under the constant fear that the same could potentially happen to you. Employee assistance programs must be on-site to offer their services, and supervisors and organizational leaders must allocate time for staff to grieve as well.

***Have socioeconomic issues placed the low-wage essential worker at greater physical risk?***

JJ: In my opinion, issues associated with some of the socioeconomic factors such as education, occupation, and income have placed low-wage workers at a higher physical risk during the COVID-19 pandemic. For instance, let us talk about the relationship between contingent work, wages, benefits, and job security. There are organizations that employ contingent workers. This practice is used to decrease payroll costs associated with providing benefits but leaves workers without basic benefits such as sick leave, healthcare coverage, and job and income stability. The unreliable number of work hours given to the contingent workers does not offer assurance that workers will be able to meet their financial commitments. The practice of hiring contingent workers also contributes to cross-organizational infection transmission as described earlier by Mr. DeWitt.

SD: I am located where housing is some of the most expensive in the United States. It is my understanding that many of the low-wage workers in the greater San Francisco area are challenged with very basic needs such an adequate, consistent home environment, some are actually homeless. The pandemic and its control measures present additional challenges related to food security, hygiene, and transportation. For example, public restrooms have been unavailable until recently, and public transportation has been reduced significantly. Restaurants are closed, many permanently, which decreases food access and possible donations for those in need. Many low-wage workers are also per diem, without health benefits, and very reluctant to test when symptomatic for fear of testing positive, which precludes them from working. All of these factors serve to place the worker at greater physical risk.

***How are companies off-setting costs of controls such as reconfiguring the workplace, distancing, partitions, and staggering work shifts?***

SS: Some agencies have received forgivable loans under the Paycheck Protection Program (PPP) that allow the organization to offset costs of labor, and use the funds to purchase personal protective equipment (PPE). Other agencies are relinquishing unneeded office space, thus saving on rent. Both of these steps allow for resources to be directed toward the essential worker.

RD: We consider group breakrooms a risk. We stagger shifts and break times. Workers are allowed to take their lunchbreaks outdoors, if weather permits. Many workers take their breaks in their private car, which we also encourage.

JJ: I agree with the strategy Dr. Sharma describes. In addition, larger organizations can repurpose their emergency funds and cover the cost of building partitions and reconfiguring workspaces to allow adequate social distancing. Furthermore, conference rooms in many organizations have been reassigned as additional breakrooms because face-to-face meetings have been replaced with virtual meetings. The cost of providing lunch meals to employees during face-to-face meetings has also been repurposed to purchase necessary partitions or additional tables and chairs in breakrooms. Employees have staggered shifts and staggered break times, when possible, minimizing the number of workers in each breakroom at a given time. Like Mr. DeWitt explained, workers were encouraged to take their lunches and breaks in designated outdoor seating areas or even in their vehicles.

***Are insurance providers working directly with organizations to prevent loss?***

VM: Yes, risk consultants are trained to engage with occupation health professionals to prevent loss. As you know, many larger manufacturing employers have on-site occupational health professionals as part of their employee health departments. The role of the insurance risk consultant is to work with the occupational health professional to mitigate unnecessary economic and human loss by identifying risk.

***Are data metrics being used to determine opportunities for risk mitigation?***

VM: Typically, a risk consultant who works as an insurance provider will begin client engagement with a comprehensive assessment of the analytics with benchmarking program performance metrics like the number of temporary total disability (TTD) days and the average cost of a TTD claim. In addition, the risk consultant will review medical management, litigation management, health benefit programs, and any specific claim trends. These trends could include incident cause, employee occupation, tenure, age stratification, and more. In addition, the risk consultant collaborates with the occupational health professional to map occupational health programs to better understand programmatic efficacy and identify gaps and inefficiencies. Data are at the heart of understanding the leading and lagging indicators that predict employee health and safety.

***Is software in place to monitor compliance with the COVID-19 transmission prevention plan?***

AR: At our organization, all team members and visitors who enter one of our facilities must fill out an electronic “Team Member COVID-19 Symptoms Monitoring Questionnaire” or the visitor equivalent. Supervisors have access to this list and verify completion to ensure compliance before each shift. Relying solely on goodwill and thinking that symptomatic or sick people will stay home is not a fail-safe option. A number of software programs are available that can perform this monitoring. When selecting a software program, consider ease of access, understandable metrics, and the ability to monitor employees effectively.

***Are low-wage workers forgotten in the COVID-19 transmission prevention plan?***

SS: I think there are challenges because the definition for low-wage workers is so broad. Low-wage workers such as housekeepers, nannies, and landscape workers are forgotten in prevention plans. More structured low-wage jobs such as grocery store or warehouse workers are included in prevention plans because they are part of larger corporate businesses and are included in an organization’s overall prevention plans.

Another challenge, particularly important to this discussion, is that health literacy is often low. This is a multilayered issue. One standard COVID-19 transmission prevention plan may not fit all. Attention to health literacy is necessary for the prevention plan to be meaningful to all employees.

Todd Mohrmann (TM): I agree with Dr. Sharma, an awareness of health literacy is really important. We know that health literacy challenges are greatest among those who are low income, those with limited education, those from minority populations, and those with limited English proficiency. Many of these same individuals are also low-wage essential workers. There are a number of tools available that organizations can use to assess their policies, practices, and print materials with respect to health literacy. Agency for Healthcare Research and Quality’s (AHRQ) “Health Literacy Universal Precautions Toolkit” and the Centers for Disease Control and Prevention’s (CDC) “Everyday Words for Public Health Communication” are both excellent resources. There are also several online text readability calculators for assessing reading level and the use of plain language in print materials. Table 1 presents a review of resources and references for the occupational health professional.

JJ: Low-wage workers in the hospital are an important part of the work flow in the care of patients with COVID-19. The hospital’s universal masking guidance considers all hospital workers who may be in direct or indirect contact with the patient. The PPE guidance provides information on the appropriate level of PPE to use depending on the type of activity being performed. The organization published a Playbook describing how to manage possible exposures in the workplace. The Playbook guides managers on the most up-to-date CDC criteria and hospital policy on timely reporting, length of time to self-quarantine and monitor, and appropriate return to work date. The Playbook is a resource that addresses the needs of all workers. Managers can adapt the Playbook to meet the learning needs of specific workers.

***What are some training ideas that can be used specifically for low-wage essential workers?***

TM: As others have said, low-wage essential workers often have more than one job and may have a greater volume of stressors outside of the workplace. Many employers, including those in healthcare and those in early childhood education, mandate training attendance for workers and are frequently unable to reduce workloads to account for time spent in training. As a result, workers often resent having to attend an educational program and receiving new information may be far from their top priority. Instructors need to empathize with these issues and ensure that training programs are engaging, high energy, hands-on, and practical. They also need to ensure that the value of the program is explicitly stated and reiterated throughout its duration—How will the information being shared help workers or make their jobs easier and safer? To the extent that instructors can articulate these benefits and also express a genuine understanding and appreciation of the workers’ job functions, they will be better able to generate participant buy-in.

TM: While there is variability in the educational levels of low-wage essential workers, as a group, they are more likely to have lower levels of educational attainment and health literacy. Instructors should use plain language in their materials and prioritize “need-to-know” versus “nice-to-know” content. In terms of presentation, content should be covered in a variety of ways to respond to different types of learners: visual, auditory, and kinesthetic. When possible, training activities should incorporate all three types of learning. For both in-person and virtual approaches, didactic segments should be limited to 15 to 20 minutes at a time. Interactive exercises should be strategically interspersed to break up lengthier didactic segments and maintain participant interest. Videos, authentic examples, visual demonstrations, case studies, and role-plays are all recommended. Obtaining real-world examples directly from participants is also a valuable strategy for maintaining engagement. Virtual training sessions should employ these same strategies to the extent possible. Maintaining engagement in virtual programs is more challenging but equally if not more important.

JJ: I agree with Mr. Mohrmann. Some, not all, low-wage workers have a lower level of formal education, which is a barrier to understanding disease prevention and transmission. This is especially true in the presence of an illness like COVID-19, which is associated with so much misunderstanding and anxiety. Understanding the disease process influences how hand hygiene, masking, and social distancing in the workplace and the home setting are practiced. Unless employees understand the risk and steps to mitigation, seldom is there consistent adherence in taking the most basic precautions.

Organizational leaders are in a position to model safe behavior. Training is important, but creating a standard of transmission precautions that is top-down can be very powerful.

***To what extent do workers resist elements of the COVID-19 transmission prevention plan?***

SS: I am not aware of any resistance to prevention plans. We have found that the motivating forces are as follows: fear of contracting COVID-19 for themselves and their families, and fear of unemployment and financial hardship among lower income families. Workers want to understand how to stay safe. I think if there is resistance, it will be to testing because there are no perceived benefits to testing. If the worker tests positive, he may stand to lose wages if quarantined. Some are in fear of losing their jobs. This is a significant challenge to mitigation strategies for COVID-19.

TM: I agree with Dr. Sharma that resistance to transmission prevention plans in and of themselves is not generally the case. My experience is that low-wage essential workers in healthcare and in early childhood education want to comply with these precautions, but organizational and management issues often get in the way. Some of these issues include the following: inadequate resource levels within facilities such as staffing and an insufficient supply of PPE, changes in messaging about precautions, inconsistent messaging about precautions throughout leadership, a haphazard organizational approach to education and training or policy implementation, and a perception that leadership does not fully understand the constraints and realities of frontline work. These are system issues that must be addressed at the leadership level to generate full compliance with safety precautions.

Workers must trust that the organization has their best interests in mind. They must feel understood and appreciated if any training is to be successful. As an example, when workloads are not reduced for a staff member’s time spent in training, the worker is more likely to feel unappreciated and resentful about being in training. Their receptivity to new information and changes in procedures is immediately compromised.

RD: We have not had workers resist changes that have been put in place. We all understand that in the dairy industry, distancing is the challenge. Working around the cattle precludes use of partitions. Employees welcome the use of PPE and it is provided by the employer.

Because of our geographic location, many workers live on-site in housing that is provided. To prevent unnecessary exposure, we assign teams comprised of (approximately) five workers who live and work together. We ask that workers enter and leave the workplace together and not socialize with others when inside or outside the workplace.

The leadership teams serve as models for transmission prevention and reinforce guidelines from the CDC. Everyone is expected to wear PPE and distance, unless the job specifically precludes this. If this is the case, other precautions are put in place such as limiting time in close proximity. We have learned from other industries, and we use these lessons to strengthen and update our response to the COVID-19 pandemic.

## Summary

Protecting the health and safety of all workers is at the heart of occupational health services and support. There is no question that individuals who live with financial insecurity are at the greatest risk for vulnerability from the COVID-19 pandemic. The COVID-19 pandemic has posed concerns and needs that would have been unforeseeable even a few years ago. The depth of financial insecurity coupled with the financial threats of the COVID-19 pandemic exacerbate social stability.

The unique needs of the low-wage, essential worker are complex. The economic elements of this situation are the basis of discussion. Lack of economic resources such as sick leave, health insurance, and life insurance are consistent, and predictable work at a living wage impacts health practices in the occupational setting. Considering the number of individuals who are categorically low-wage essential workers, opportunity exists to explore better ways to protect workers, their families, and employers in the presence of the COVID-19 pandemic. Limitations to this article include the rapidly evolving nature of the COVID-19 pandemic, and the discussion did not include the opinions of low-wage, essential workers.
